# Rumination and Age: Some Things Get Better

**DOI:** 10.1155/2012/267327

**Published:** 2012-02-22

**Authors:** Stefan Sütterlin, Muirne C. S. Paap, Stana Babic, Andrea Kübler, Claus Vögele

**Affiliations:** ^1^Research Unit INSIDE, University of Luxembourg, Campus Walferdange, Route de Diekirch, 7220 Walferdange, Luxembourg; ^2^Research Group on Health Psychology, University of Leuven, Tiensestraat 102, 3000 Leuven, Belgium; ^3^Department of Research Methodology, Measurement and Data Analysis, Faculty of Behavioral Sciences, University of Twente, Drienerlolaan 5, 7522 NB Enschede, The Netherlands; ^4^Department of Psychology I, University of Würzburg, Marcusstraße 9-11, 97070 Würzburg, Germany

## Abstract

Rumination has been defined as a mode of responding to distress that involves passively focusing one's attention on symptoms of distress without taking action. This dysfunctional response style intensifies depressed mood, impairs interpersonal problem solving, and leads to more pessimistic future perspectives and less social support. As most of these results were obtained from younger people, it remains unclear how age affects ruminative thinking. Three hundred members of the general public ranging in age from 15 to 87 years were asked about their ruminative styles using the Response Styles Questionnaire (RSQ), depression and satisfaction with life. A Mokken Scale analysis confirmed the two-factor structure of the RSQ with brooding and reflective pondering as subcomponents of rumination. Older participants (63 years and older) reported less ruminative thinking than other age groups. Life satisfaction was associated with brooding and highest for the earlier and latest life stages investigated in this study.

## 1. Introduction

Repetitive thoughts have been defined as the “process of thinking attentively, repetitively or frequently about one's self and one's world” [1, page 909]. Their constructive properties are discussed in terms of enhanced adaptive preparation, anticipatory planning, and others (for an overview see [2]). Amongst repetitive thoughts, those of a ruminating style characterized by depressive contents (depressive rumination) are seen as particularly unconstructive and maladaptive. Depressive rumination has been defined in various ways (see [3]), with one of the most frequently used provided by Nolen-Hoeksema and colleagues [4] who conceptualize depressive rumination as “behaviors and thoughts that focus one's attention on one's depressive symptoms and on the implications of these symptoms” [4, page 569]. Using this definition, the current paper investigates depressive rumination as a risk factor for depression and a personality trait that differentiates between healthy individuals and their individual levels of nonclinical depression.

The concept of depressive rumination was introduced several years ago within the framework of Nolen-Hoeksema's Response Styles Theory (RST, [5–7]). Ruminators tend to remain fixated on the problems and on their feelings about them without taking action [4]. Previous research suggests that depressive rumination intensifies depressed mood and predicts the onset, recurrence, severity, and duration of depressive episodes [8, 9]. Ruminators are ineffective in active, interpersonal problem solving [10, 11], and a depressive ruminative response style leads to more pessimistic future perspectives and less social support [12, 13]. For example, in a recent study [14], rumination was shown to foster indecision, indicating that rumination interferes with decision making in dysphoric individuals. In addition, depressive rumination has been demonstrated to mediate several other hypothesized risk factors that prospectively predict the number of depressive episodes, including dysfunctional attitudes, neediness, self-criticism, and history of past depression [15]. In a sample of patients with fatal neurodegenerative disease, depressive rumination was found to mediate the negative effect of reminiscence on well-being [16]. This suggests that a ruminative response style is an important factor for both the onset and maintenance of depression, and that it is an important target for treatments aimed at reducing current and future affective symptoms.

Previous research on ruminative thinking has mainly focused on affective components of well-being, such as depression. Beyond affect, a distinct cognitive component of subjective well-being has been referred to as life satisfaction [17]. Life satisfaction refers to a global, cognitive-judgmental aspect of a person's life, based on one's own standards, goals, and weightings of various life domains, and plays a complementary role for the judgment of subjective well-being [18, 19]. Life satisfaction is negatively correlated with depression [20, 21] but is more sensitive to change and shows some degree of independence from purely affective constructs, including depression [19]. Given that both depression and life satisfaction are known to change over the lifespan and are associated with depressive rumination, this triangular relationship needs to be investigated with regard to age-related effects. To our knowledge, the relationship between ruminative response styles and global life satisfaction has not yet been investigated with respect to the influence of aging. Considering the partial independence of the purely affective construct of depression and the more cognitive-judgmental construct of life satisfaction, the current study aimed at investigating specific influences of ruminative styles on both measures of subjective well-being.

Studies investigating rumination on the background of the RST have typically used the 22-item Rumination Reponse Scale (RRS) of the Response Style Questionnaire (RSQ, [6]). Treynor and colleagues [22] postulated two factors to further differentiate the construct of rumination. Their results support a two-factor model of rumination, which includes the components of reflective pondering and brooding, the latter of which was found to be the key factor in the prediction of depressive symptoms. Brooding “relates to passive and self-critical thoughts comparing one's current situation against a desired standard or goal” [23, page 605], whereas reflection “refers to a more purposeful inward examination and attempt at problem-solving in response to depressed mood” (page 605, [22, 23]). Armey and colleagues [24] replicated these results such that they identified the same two-factor structure as Treynor and colleagues [22] with brooding being more strongly related to depression than pondering.

Taken together these results provide accumulating evidence that rumination is a key factor in depression. Nevertheless, studies investigating the association between rumination, depression, and life satisfaction over the lifespan are rare. The majority of studies, which address the two postulated components of rumination, either examined primarily undergraduate students [24] or did not address age as a variable under investigation [22], although clinical and epidemiologic findings suggest that depression and life satisfaction are age dependent.

Adolescence, for example, has been repeatedly identified as a critical period in the lifespan for the onset of a range of mental disorders, including depression and anxiety. The majority of results show a peak in adolescence and a subsequent decline in incidence and prevalence for most mental disorders with increasing age [25–27]. The reasons for these associations have been discussed in terms of changing life circumstances, critical life events, and other external factors determining the presence of stressors [27], as well as neurodevelopmental causes [28, 29]. Other factors for these age-dependent effects may be explained by findings indicating decreased emotional responsiveness with age, increased emotional control, and psychological immunization to stressful experience. Based on these results, we hypothesize that rumination, which is known to be associated with the occurrence of depression, to be more pronounced in early adulthood, and to be lowest in older adults.

At the other end of the lifespan and supporting the hypothesis of an inverse association between aging and rumination, studies examining age differences in emotional experiences have dispelled the myth of age-related decline of well-being. For decades, this decline was taken as common sense [30], probably caused by assumptions based on the decline in physiological functions and the increase in negative life events such as loss of friends. Older age is not associated with increased emotional distress [31]; to the contrary, there is even a slight decrease in self-reported negative affect in older adults compared to middle-aged and younger adults [32], as well as lower rates of anxiety and major depression [33, 34]. A steady decline in subclinical depression has been reported across young, middle and older adulthood [35]. In terms of lifetime prevalence, 1% of older adults are diagnosed with major depression, compared to 6% of younger adults [36]. With the exception of a slight age-related increase in depressive symptoms reported in some studies, the majority of studies found depression levels to be lower in older compared to younger adults [31, 33]. It has to be conceded, however, that a decreasing prevalence of major depression with age is not equivalent to decreased depression levels per se, as minor, subsyndromal depression is usually not caught by studies focusing on major depression [37]. The current study will thus discuss depression scores obtained using a self-report questionnaire designed for nonclinical populations. A better understanding of the age-dependent associations of reflecting, brooding, depression and life satisfaction in a nonclinical sample may help in the prevention of mental ill-health and contribute to the development of cohort-specific therapeutic interventions.

In the current study, first, a replication of Treynor and Armey's [22, 24] two-factor structure was conducted to ensure the implementation of identical factors as described in previous research. This attempted replication was based on the Response Style Questionnaire's (RSQ) 10-item rumination subscale, as extracted from Treynor and colleagues [22]. Second, we hypothesized that depressive rumination would be most pronounced at a younger age and would be lowest in the oldest investigated age group. Third, we explored the relative contribution of the different ruminative styles of brooding and reflection to affective (i.e., depression) and cognitive-judgmental aspects (i.e., life satisfaction) of subjective well-being.

## 2. Methods

### 2.1. Participants

The sample consisted of 300 members of the general public. They were approached in public settings (e.g., cafes, retirement communities, long distance trains, public squares) using ad hoc recruitment. Data from one person was excluded from analysis due to incomplete sociodemographic information. The remaining 299 participants (118 women) were aged between 15 and 87 years (M = 41.90, SD = 18.57). A total of 269 (89.7%) participants were native German speakers, the remaining 31 (10.3%) participants spoke fluently German. One hundred and eleven (37.0%) participants were single, 76 (25.3%) were living in a partnership, 78 (26%) were married, 22 (7.3%) were divorced, and 13 (4.3%) widowed. A total of 89 persons (30.1%) completed secondary school, 173 participants (58.4%) achieved a university entrance qualification.

### 2.2. Age Categories

Assignment to age categories followed the World Health Organization's definition of “youth” encompassing the age range of 15–24 [34]. “Seniors” were defined as 63 years of age or older, following the actual pensionable age in Germany according to the national statistics office (http://www.destatis.de/). Participants with an age between 24 and 63 years were divided into three groups with approximately equal age ranges of 12 and 11 years.  Table 1 shows proportions of participants in each age category together with their mean age and standard deviation.

### 2.3. Measures

Rumination style was assessed with Treynor and colleagues' [22] version of the Response Style Questionnaire (RSQ), which assesses ruminative styles on the subscales brooding (Cronbach's alpha =  .72) and reflective pondering (Cronbach's alpha =  .60). Both subscales consist of five items to be answered on a 4-point Likert-scale (e.g., brooding: “What am I doing to deserve this?” or reflective pondering: “I analyze recent events and try to understand why I am depressed.”). The original version was translated and backtranslated by the authors. The German translation applied in this study reached comparable internal consistency (Cronbach's alpha =  .69 for subscale brooding; Cronbach's alpha =  .75 for subscale reflective pondering).

Depression was assessed with the Allgemeine Depressivitäts-Skala Langversion (ADS-L, [38]), which is the German version of the Center of Epidemiologic Studies Depression Scale (CES-D; [39]). The CES-D was developed to assess depressive symptoms in nonclinical populations. With its high sensitivity, the questionnaire is suitable to assess interindividual differences in depression in highly functional, nonclinical samples. A total score is calculated from 20 items to be rated on a 4-point Likert-scale (e.g., “I could not get going,” “I felt lonely”). The CES-D focuses on current states. In representative nonclinical samples, split-half reliability (*r* = .89) and test-retest reliability (*r* =  .45–.70, depending on the time interval) were reported as excellent and very good, respectively [39]. Psychometric properties of the German translation have been reported to be good [38].

Satisfaction with life was assessed using the Satisfaction With Life Scale (SWLS; [18]). The SWLS assesses cognitive-judgmental aspects of subjective well-being based on a standard that each individual sets for him or herself. Five items are responded to on a 6-point Likert-scale; scores can range from 5 (low satisfaction) to 30 (high satisfaction) points (e.g., “In most ways my life is close to my ideal,” “If I could live my life over, I would change almost nothing”). The two-month test-retest correlation coefficient was  .82, and Cronbach's alpha was  .87 [18].

### 2.4. Factor Replication

#### 2.4.1. Mokken Scale Analysis

A factor replication was carried out to validate the German translation of the RSQ and to ensure that the factor structure reported in the original version is independent of age. To investigate the dimensionality of the Rumination scale, Mokken Scale Analysis (MSA) was used. Mokken Scale Analysis is a form of nonparametric item response theory (NIRT), which consists of a family of measurement models that are based on a minimal set of assumptions; making them especially appropriate for the scale analysis of ordinal data such as rating scales [40–43]. Mokken Scale Analysis was applied using the software package Mokken Scale Analysis for Polytomous items (MSP5.0, [44]).

We used the Double Monotonicity Model (DMM), which is based on four assumptions: unidimensionality, local independence, monotonicity, and double monotonicity. *Unidimensionality*: the items measure one latent trait only (referred to as **θ**). *Local independence*: the scale consists of items which the participant approaches in a way that is independent of the previous items. *Monotonicity*: the Item Response Functions (IRFs) should be monotone nondecreasing (monotonicity). This means that an increase in **θ**-level never corresponds with a decrease in the probability of choosing item category *m* or higher. Together, these three assumptions result in a measurement model which can be used to rank-order *respondents* on an underlying unidimensional scale using the unweighted sum of item scores [42, 45–47]. 

#### 2.4.2. Double Monotonicity

It is assumed that the I(S)RFs (see endnote^1^) do not intersect across the latent trait. This assumption holds if there is an unambiguous rank ordering of items and response categories within each item. If this assumption holds, the items can be unambiguously ordered on the underlying trait [42, 43].

In order to evaluate whether the scale or scales are unidimensional, scalability coefficients are calculated. These coefficients are calculated between item pairs (*H*_*ij*_), on the item level (*H*_*i*_), and on the scale level (*H*). *H*_*ij*_ equals the items' covariance corrected for the maximum covariance given the items' univariate score-frequency distributions [48]. An important advantage of this statistic is that it avoids problems with respect to the distorting effect of difference in item-score distributions on inter item correlations [47]. The *H_i_*s are based on the *H_ij_*s and express the degree to which an item is related to other items in the scale. *H* is based on the *H_i_*s and expresses the degree to which the total score accurately orders persons on the latent trait scale. A scale is considered acceptable if  .3 ≤ *H* < 0.4, good if  .4 ≤ *H* < .5, and strong if *H* ≥ .5 [42, 49].

MSP5.0 offers the possibility to perform an exploratory or confirmatory analysis. In this study, we chose the confirmatory analysis (option “TEST” in MSP5.0). We tested two scale solutions: one assuming a unidimensional structure (rumination), and one assuming a two-dimensional structure (brooding and reflection). In the first analysis, all RSQ items were entered. In the second analysis, the two subscales were tested separately. These two analyses were then compared and the best solution was chosen based on the *H*-values and the aforementioned assumptions. Testgraf98 [50] was used to produce the IRFs.

### 2.5. Statistical Analysis

A one-way between groups multivariate analysis of variance was performed to investigate group effects of age on ruminative style. The two dependent variables were the subscale brooding of the RSQ and subscale reflective pondering of the RSQ. The independent variable was age (five levels, see Table 4). Preliminary testing checked for normality, linearity, univariate and multivariate outliers, homogeneity of variance-covariance matrices, and multicollinearity, with no serious violations noted. In a second step, a regression analysis was used to model the relationship between the independent variables reflective pondering/brooding and the dependent variables depression/life satisfaction, while controlling for sex and age and investigating all interaction effects between sex, age, and the two rumination subscales. Separate regression analyses were used for depression and life satisfaction. For factors sex and age, the reference categories used were *male* and *age* between 25–37 years, respectively. To test whether the relationship between the independent variables and dependent variables was dependent on sex or age group, interaction effects were calculated. The models were built using a forward procedure, adding one variable at a time. We started with the control variables sex and age and continued by adding brooding, reflective pondering, and the interaction effects. Interaction effects were not included in the final model if they were not significant. All regression analyses were performed in PASW Statistics 18.0.2 (PASW, 2010). An alpha of 0.05 was used.

## 3. Results

### 3.1. Descriptive Statistics

Self-report measures were within normal range (Table 2) compared with previous reports [18, 22, 38].

### 3.2. Factor Replication

#### 3.2.1. Mokken Scale Analysis

Two confirmatory analyses were conducted. In analysis 1, all items were included in one scale, whereas analysis 2 tested the two hypothesized subscales of rumination (i.e., brooding and reflective pondering). As can be seen from Table 3, both analyses resulted in scale-*H* values exceeding 0.3, implying that both scale solutions could be considered acceptable. However, closer inspection of the individual item-*H* (*H*_*i*_) values revealed that 5 items had *H*_*i*_ values lower than 0.3 for the unidimensional model. This was not the case in the second analysis: here, all *H*_*i*_ values exceeded 0.3. Therefore, the 2-scale solution was deemed superior. Tests for monotonicity available in MSP5.0 indicated that this assumption was possibly violated for item 10. However, visual inspection of the summary IRF for this item, using TestGraf98, did not show any violation. The checks for double monotonicity did not show any violations. Furthermore, the item ordering within the two subscales was comparable for the five age groups.

**Figure 1:**
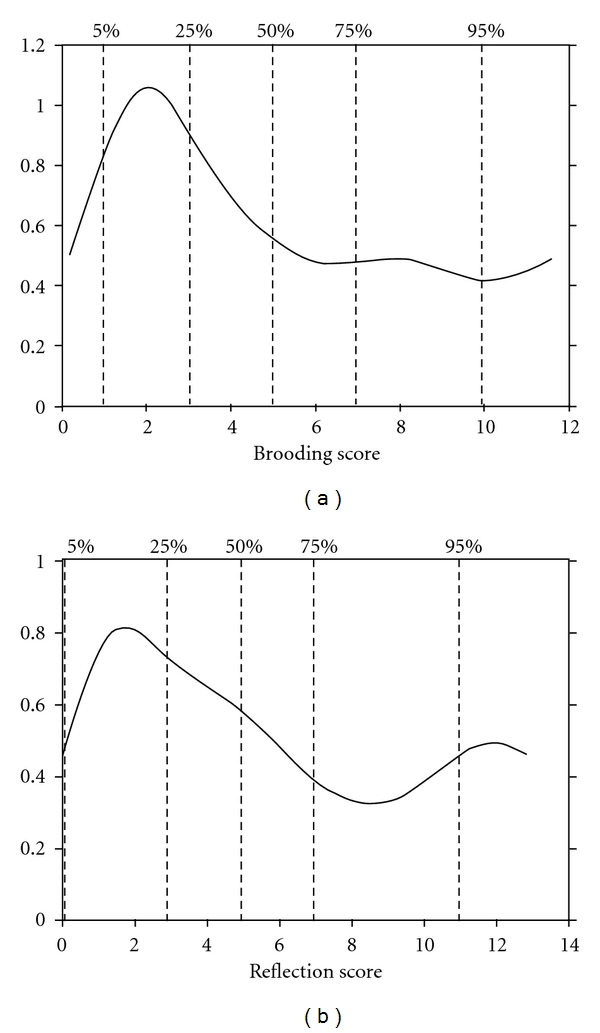
Test information function for the brooding subscale (a) and the reflection subscale (b), with estimated scale scores on the horizontal axis and test information on the vertical axis. *Note:* the vertical dashed lines indicate (from left to right) the 5th, 25th, 50th, 75th, and 95th percentiles of the observed score distribution, respectively.

Figure 1 presents the information functions of the brooding and reflection subscales. These curves depict the measurement precision for a person with a given score on the latent trait scale (**θ**), which is estimated by the expected total score. The graphs clearly show that the measurement precision for the two subscales is best at the lower levels of expected total score. This observed difference of information at different levels of **θ** suggests that the two rumination subscales cannot distinguish reliably between persons with moderately high and very high scores on the brooding and reflection subscales.

Taken together, the factor structure reported in the English language version of Treynor and colleagues [22] has been replicated in the German translation and was independent of age.

### 3.3. Multivariate Analysis of Variance

#### 3.3.1. Age Differences in Ruminative Style

Significant age effects were found for both the brooding subscale (*F*(4,293) = 5.63, *P* = .000, partial *η* ^2^ = .07) and the reflective pondering subscale (*F*(4,293) = 5.93, *P* = .000, partial *η* ^2^ = .08). Post hoc comparisons using Tukey's HSD test with an adjusted alpha level of  .01 indicated that, on the reflective pondering subscale the oldest age group (≥63 years) compared to the three age groups ≤24 years (*d* = .77), 25–37 (*d* = .76), and 38–50 (*d* = .85) showed lower scores (*P* < .001) and marginally significant lower scores compared to the second oldest age group of 51–62 years (*d* = .59). Scores on the brooding subscale indicated statistically significant differences between the oldest age group (≥63 years) and the youngest age group (≤24 years, *d* = .37) only. For both, brooding and reflective pondering subscales, there were no significant differences between the other age groups. Self-reported data are given in Table 4. Overall, the oldest age group reported less ruminative thoughts, which was expressed in medium to large effect sizes in comparison to younger age groups.

### 3.4. Regression

#### 3.4.1. Association between Ruminative Style and Depression

The final model for depression is presented in Table 5. Brooding had a positive significant association with depression, which did not depend on age or sex. In contrast, the association of reflective pondering was dependent on age and sex. The significant three-way interaction between age, reflective pondering, and sex indicates that the association between reflective pondering and depression was different for age, but only for one of the two sexes. Since men were used as the reference category, the significant two-way interaction between reflective pondering and age indicates that for men up to 24 years and 63 years and older, the association between reflection and depression was stronger than for men at the age of 25 to 37 (positive value of standardized beta). For women, however, no such age effects were found. This is indicated by the significant three-way interaction of age, reflective pondering, and sex, which has a negative standardized beta value of approximately the same size as the positive value of the two-way interaction.

#### 3.4.2. Association between Ruminative Style and Life Satisfaction

The final model for life satisfaction is presented in Table 5. Reflective pondering was not associated with life satisfaction and was, therefore, not included in the final model. Brooding had a negative significant association with life satisfaction. This effect was weaker for age groups 38–50 years and 51 to 62 years compared to group 25 to 37 years, which is indicated by a significant negative standardized beta value for the two-way interactions between brooding and respective age groups. Significant main effects were also found for these age groups, indicating that participants aged 38 to 62 experienced a lower life satisfaction than participants aged 25 to 37, regardless of sex or brooding.

## 4. Discussion

To our knowledge, the present study is the first to investigate depressive ruminative response styles in different age groups in a cross-sectional design in general, and the association of rumination with depression on the one hand, and life satisfaction on the other. By comparing age groups this study builds upon previous work investigating the presence of perseverative thinking in exclusively young or old samples [23] and previous research replicating the association between rumination and depression in older samples without differentiating between ruminative styles [51]. Up to now life satisfaction as a cognitive judgment of well-being in relation to one's own individual standards has not yet been investigated in relation to ruminative response styles. Prior to further analyses, however, a German translation of the 10-item version of the RSQ [22] was subjected to a nonparametric Mokken scale analysis based on item response theory, which confirmed the factor assignment as previously reported by Treynor and colleagues [22]. The factor assignment was identical in all age groups, the item-ordering in the MSA comparable, demonstrating that the 10-item version of the RSQ is adequate for younger and older samples alike.

Results of the present study are in line with numerous earlier findings suggesting a stable association between ruminative response style and depression (e.g., [6, 7]). As expected, this association was significantly stronger for the subscale brooding as compared to reflective pondering and most pronounced for the youngest and the oldest age group. The oldest age group showed remarkably low indicators of rumination (i.e., low brooding and low reflective pondering sores).

Life satisfaction was not affected by reflection but by brooding. This association was pronounced in the youngest and oldest age group, supporting the concept of separate cognitive mechanisms for brooding and reflective pondering. Effects of age categories indicated a high burden of ruminative brooding in the youngest age group. This finding was accompanied by high depression scores in young people, although their life satisfaction was not negatively affected in comparison to other age groups. Notably, reflective pondering in the oldest and youngest age group of men was more strongly associated with depression than for their female counterparts. Previous results suggest higher levels of ruminative behavior in women compared to men [4, 5, 7], accounting for sex differences in the prevalence of major depression. The present study suggests that the association between pondering and depression is not necessarily absent in male samples but depends on the life period in which it occurs. The reasons for age- and sex-dependent associations between reflective pondering and depression have to remain unanswered at this point. A promising approach could be to investigate age-dependent topics of reflection or brooding that might differ between the sexes. We suggest that different periods over the life span and their particular cohorts are confronted with age-specific life events, challenges, and opportunities. Qualitative research approaches might contribute to understand these differences better. Future research should also investigate potential determinants of the varying association between reflective pondering and depression at different life stages, for example, the actual content of reflection, physical and mental health status, and so forth. It could be speculated, for example, that the shorter life expectancy of men together with the increased likelihood of health complaints in the oldest age group might change the content of reflective pondering towards more health-related thinking, thus increasing the likelihood of sex differences to emerge. However, further speculations on the underlying processes and mechanisms accounting for these effects are beyond the scope and possibilities of this cross-sectional study.

These results have implications for cognitive therapies with older patients. Whereas the special needs of young and adolescent persons have been discussed for many years and are now included in the curricula of clinical trainings, the specific requirements of psychological interventions for older people have only recently started to be taken into consideration. Research on “good aging” [52–55] and its clinical consequences for cognitive behavioral psychotherapy as well as the CCMSC-model (“context, cohort, maturation, & specific challenges”; [56]) are examples of this ongoing development of paradigms. The different ruminative behavior as reflected in the current self-reports may be due to the fact that cohorts are raised in specific historical contexts and therefore in their particular social and societal environments. These contextual effects might influence developmental processes at cognitive and metacognitive levels. The further development of cohort-specific intervention requires a sound understanding of developmental aspects of cognitive function, particularly where risk factors for mood disorders are concerned. The present study contributes to this understanding in a way that ruminative behavior appears to be less pronounced in older adults beyond retirement age and that this ruminative style is associated with depression and negative affect to a lesser extent, as it is at a younger age. Further research is needed to compare these findings with adult clinical samples or vulnerable groups with specific physical or psychological impairments and to identify the mechanisms underlying these stabilizing and protective processes. These processes have been discussed in terms of goal adjustment and their age-related shifts [52, 54]. At a stage of life when irreversible and uncontrollable events accumulate, accommodation of goals and a different weighing of personal priorities have been shown to provide the basis for the remarkable stability, resourcefulness, and resilience of aging adults [57].

One limitation of this study is the lack of health-related information that might have influenced the responses. It was seen as not appropriate to ask personal questions regarding one's health in the various settings. Thus, it cannot be ruled out that fidelity of answering personal questions and social desirability brought forward in a questionnaire delivered by an unknown person is also influenced by the age or sex of the participant as well as the researcher and that these experimenter effects are age specific. Nevertheless, higher age is more likely to be associated with ill health, and thus health-related rumination would have worked against the hypothesis of lower rumination scores in high age. The fact that this study is cross-sectional is seen as providing important information about possible, although not quantifiable, cohort effects that are expected to exist and to imply differential consequences for therapeutic approaches. Another limitation concerns the ad hoc recruitment of participants. It cannot be ruled out that the particular settings where participants were recruited for the current study implied certain self-selection biases. Nevertheless, a certain selection bias is unavoidable in studies investigating “representative” samples of the population at large, who do not have the possibility to draw a balanced sample (e.g., with the help of public authorities or opinion poll agencies) but recruit participants in a random fashion. Subsequent studies should replicate the present results in other samples, or particularly chosen subsamples (e.g., age groups).

## 5. Conclusions

The presented results provide a replication of Treynor's and Armey's two-factor structure of a German translation of the RSQ's 10-item rumination subscale. The dimensionality of brooding and reflecting could be confirmed in a Mokken Scale analysis based on nonparametric item response theory modeling, supporting earlier notions of distinct cognitive processes for reflective pondering and brooding. Previous findings on a positive relationship between depression and rumination were confirmed, as was the particular close association between brooding and depression. This association was most pronounced for the youngest and oldest age group, although the absolute values indicated a relatively lower burden of rumination in the oldest age group. Life satisfaction was associated with brooding but not with reflective pondering.

To our knowledge, this study is the first to investigate the association of ruminative styles and depression in different age groups. The findings are in line with recent research on successful aging and data on vulnerability for psychological problems in adolescents. The results suggest further that more research is needed on age-dependent effects of ruminative styles and their potential consequences for clinical psychological assessment and intervention. More generally, this study aims to inspire further research including comparisons of various life stages and requires more detailed investigations of the underlying mechanisms, particularly of age by sex interactions, which were beyond the possible scope of the present study. Given the rather low scores of rumination in the older age group, it could be speculated, for example, that interventions designed to reduce depression in older age might be more effective if they focused on behavioral activation rather than cognitive restructuring. Moreover, results on life-satisfaction indicated age-dependent associations between ruminative styles and satisfaction with life on the one hand and depression on the other.

## Figures and Tables

**Table 1:** Age categories and descriptive statistics.

Age	*n*	Percent	M	SD
≤24 years	65	21.7	21.08	2.36
25–37 years	80	26.8	28.90	3.35
38–50 years	50	16.7	43.62	4.03
51–62 years	44	14.7	56.11	3.58
≥63 years	60	20.1	69.92	6.29
Total	299	100.0	41.90	18.57

**Table 2:** Self-report measures across the whole sample.

	*n*	Min	Max	M	SD
RSQ					
Brooding	298	0	26	5.08	2.60
Reflective pondering	298	0	14	5.04	3.12
ADS-L	298	0	46	13.63	8.69
SWLS	297	0	35	25.31	5.47

RSQ: Response Style Questionnaire; ADS-L: Allgemeine Depressivitätsskala Langversion (German version of the Centre of Epidemiological Studies Depression Scale); SWLS: Satisfaction With Life Scale.

**Table 3:** Item means.

	Analysis 1		Analysis 2	
	Item mean	*H*_*i*_	*H*_*i*_ (Scale “B”)	*H*_*i*_ (Scale “R”)
5. “What am I doing to deserve this?”	0.71	0.24	0.32	
7. Analyze recent events	1.38	0.27		0.47
10. “Why do I always react this way?”	1.25	0.35	0.32	
11. Go away by yourself	1.29	0.35		0.44
12. Write down what you are thinking	0.42	0.25		0.37
13. Think about a recent situation	1.25	0.27	0.43	
15. “Why do I have problems other people do not have?”	0.75	0.27	0.41	
16. “Why cannot I handle things better?”	1.11	0.42	0.42	
20. Analyze your personality	1.04	0.42		0.49
21. Go someplace alone to think about your feelings	0.91	0.30		0.43

*H* (total scale)		0.33	0.38	0.44

Rho*		0.79	0.71	0.76

*Reliability estimate provided by MSP5.0; B: brooding; R: reflection.

*Note. *Item numbers refer to the 22-item version of the Ruminative Response Scale [22] after exclusion of items loading on the factor depression.

**Table 4:** Self-report measures by age groups.

Age group	≤24		25–37		38–50		51–62		≥63	
	*n*	M (SD)	*n*	M (SD)	*n*	M (SD)	*n*	M (SD)	*n*	M (SD)
RSQ reflective pondering	65	5.40 (2.76)	80	5.62 (3.40)	50	5.54 (2.56)	44	5.11 (3.47)	59	3.36 (2.72)
RSQ brooding	65	4.54 (1.95)	80	3.85 (2.19)	50	3.66 (2.12)	44	4.18 (2.19)	59	2.88 (1.94)
CES-D	65	14.98 (9.46)	80	13.99 (9.10)	50	12.98 (8.10)	44	12.41 (6.21)	59	13.10 (9.33)
SWLS	65	25.69 (5.71)	79	25.52 (6.14)	50	25.28 (5.02)	44	23.66 (5.46)	59	25.85 (4.48)

**Table:** (a) Effects of age, sex, brooding and reflection on depression

	Unstandardized coefficients		Standardized coefficients	Sig.	95.0% confidence interval for *B*		*R* ^2^
	*B*	Std. Error	Beta	*P*	Lower bound	Upper bound	
Sex	−2.088	3.611	−.118	.564	−9.197	5.021	0.052
Age group 1 (≤24 y.)	−4.588	3.622	−.218	.206	−11.718	2.541	0.123
Age group 3 (38–50 y.)	.781	4.738	.034	.869	−8.547	10.109	
Age group 4 (51–62 y.)	−4.683	5.278	−.192	.376	−15.074	5.708	
Age group 5 (≥63 y.)	−4.218	3.374	−.193	.212	−10.860	2.424	
IA sex × age group 1	7.376	5.544	.284	.184	−3.538	18.291	0.202
IA sex × age group 3	1.131	6.499	.040	.862	−11.664	13.925	
IA sex × age group 4	7.183	6.490	.263	.269	−5.594	19.960	
IA sex × age group 5	6.428	4.920	.255	.193	−3.258	16.113	
RSQ subscale brooding	1.375	.197	.411	.000	.986	1.763	0.259
RSQ subscale reflective pondering	−.053	.327	−.019	.871	−.697	.591	0.271
IA reflective pondering × age group 1	1.429	.673	.423	.035	.103	2.754	0.287
IA reflective pondering × age group 3	.168	.719	.045	.816	−1.248	1.584	
IA reflective pondering × age group 4	.882	.893	.228	.324	−.876	2.640	
IA reflective pondering × age group 5	2.135	.778	.438	.006	.603	3.668	
IA sex × reflective pondering	.956	.529	.387	.072	−.086	1.997	0.288
IA sex × age group 1 × reflective pondering	−2.220	.906	−.570	.015	−4.003	−.436	0.312
IA sex × age group 3 × reflective pondering	−1.048	1.009	−.215	.300	−3.034	.939	
IA sex × age group 4 × reflective pondering	−1.933	1.048	−.450	.066	−3.997	.130	
IA sex × age group 5 × reflective pondering	−2.255	.981	−.409	.022	−4.187	−.323	

IA: interaction, refl.: reflective pondering. Interaction effects were not included in the final model if they were not significant; R^2^ is reported for the model containing the particular variable and all variables listed before that variable.

**Table:** (b) Effects of age, sex, brooding, and reflection on life satisfaction

	Unstandardized coefficients		Standardized coefficients	Sig.	95,0% Confidence interval for *B*		*R* ^2^
	*B*	Std. error	Beta	*P*	Lower bound	Upper bound	

Sex	.604	.608	.054	.322	−.593	1.801	0.008
Age group 1 (≤24 y.)	−.627	2.078	−.048	.763	−4.718	3.464	0.136
Age group 3 (38–50 y.)	−4.194	2.008	−.288	.038	−8.147	−.241	
Age group 4 (51–62 y.)	−5.635	2.108	−.367	.008	−9.785	−1.485	
Age group 5 (≥63 y.)	−2.945	1.760	−.214	.095	−6.411	.520	
RSQ Subscale Brooding	−1.336	.221	−.633	.000	−1.772	−.900	0.451
IA brooding × age group 1	.295	.342	.144	.389	−.379	.969	0.471
IA brooding × age group 3	.756	.347	.306	.030	.072	1.439	
IA brooding × age group 4	.729	.354	.290	.040	.032	1.426	
IA brooding × age group 5	.383	.349	.129	.273	−.304	1.070	

IA: interaction, refl.: reflective pondering. Interaction effects were not included in the final model if they were not significant; *R* ^2^ is reported for the model containing the particular variable and all variables listed before that variable.

## Endnotes

1. The basic unit in any item response theory (IRT) model is the item response function (IRF; also known as the item characteristic curve, ICC). In case of dichotomous items, the IRF depicts the relationship between the latent trait **θ** (*x*-axis) and the probability of the item being endorsed (*y*-axis). The term “latent” is used because the trait cannot be observed directly but can only be inferred from other variables (items in the test). An IRF can still be produced for polychotomous data but is now the sum of the so-called item step response functions (ISRFs). The ISRF could be seen as a special case of the IRF, depicting the probability of answering in category *m* or higher. Since the probability of answering “at least” in the lowest category is equal to 1, we are left with (*m* − 1) ISRFs for each item. In our case, there were 4 answering categories; hence, the number of ISRFs per item were 3.
2. The concept of information is related to the concept of reliability; both are indicators for the quality of the test. Information has the advantage that it is not dependent on population heterogeneity. Modern test theory (IRT) offers the advantage that reliability as well as information can be measured as a function of the latent trait.
